# Data for designing two isothermal amplification assays for the detection of root-infecting fungi on cool-season turfgrasses

**DOI:** 10.1016/j.dib.2018.08.021

**Published:** 2018-08-17

**Authors:** Brijesh B. Karakkat, Kurt Hockemeyer, Margot Franchett, Megan Olson, Cortney Mullenberg, Paul L. Koch

**Affiliations:** Department of Plant Pathology, University of Wisconsin-Madison, 1630 Linden Drive, Madison, WI, USA

**Keywords:** Turfgrass, LAMP, RPA, *Gaeumannomyces avenae*, *Ophiosphaerella korrae*, *Magnaporthiopsis poae*

## Abstract

Loop-mediated isothermal amplification (LAMP) and recombinase polymerase amplification (RPA) are two rapid isothermal amplification methods for detecting three common fungal root pathogens of cool-season turfgrass: *Gaeumannomyces avenae*, *Magnaporthiopsis poae* and *Ophiosphaerella korrae, “*Detection of root-infecting fungi on cool-season turfgrasses using loop-mediated isothermal amplification and recombinase polymerase amplification*”* (Karakkat et al., 2018) [1]. The data provided here describe the information for designing primers and probes for LAMP and RPA, how specific they are for each of the three fungi, and the evaluation of RPA on field samples.

**Specifications Table**

**Table:** 

Subject area	*Biology*
More specific subject area	*Plant pathogen detection*
Type of data	*Tables and Figures*
How data was acquired	*Gel electrophoresis, BLAST tools, RPA detection*
Data format	*Raw, analyzed*
Experimental factors	*Three root-infecting fungi- Gaeumannomyces avenae*, *Magnaporthiopsis poae* and *Ophiosphaerella korrae and turf root samples showing symptoms of any of those three fungal infections.*
Experimental features	*Specificity of LAMP primers and RPA primer and probes, RPA evaluation on field samples*
Data source location	*Madison, Wisconsin, USA 43.0731°N, 89.4012°W*
Data accessibility	*The data are available with article*
Related research article	*Karakkat, B.B., Hockemeyer, K., Franchett, M., Olson, M., Mullenberg, C., Koch, P.L, Detection of root-infecting fungi on cool-season turfgrasses using loop-mediated isothermal amplification and recombinase polymerase amplification. Journal of Microbiological Methods, (2018) 151:90–98*[1]

**Value of the data**

• This article provides data for the design of primers and probes of the two isothermal detection methods Loop-mediated isothermal amplification (LAMP) and recombinase polymerase amplification (RPA) for detecting three important turf root-infecting fungi namely *Gaeumannomyces avenae*, *Magnaporthiopsis poae* and *Ophiosphaerella korrae.*
• The article has gene sequence information for the three fungal pathogens of cool-season turfgrasses that can be used to design LAMP primers and RPA primers-probes by plant diagnosticians, turf superintendents, athletic turf field managers to design and conduct same assays in their diagnostic facilities.
• The technical information of LAMP and RPA can also be used by researchers and diagnosticians alike to employ similar design approaches for other fungal pathogens of cool-season and warm-season turfgrasses that are difficult to diagnose by traditional methods of microscopy and culturing.

## 1 Data

The data includes the design of the LAMP primers and RPA primers-probes sequences that were tested for specificity between the three fungal pathogens. The RPA assay was evaluated on 2016 field samples received at turfgrass diagnostic laboratory, Madison, Wisconsin, USA.

### 1.1 LAMP and RPA specificity

**Fig. 1:**
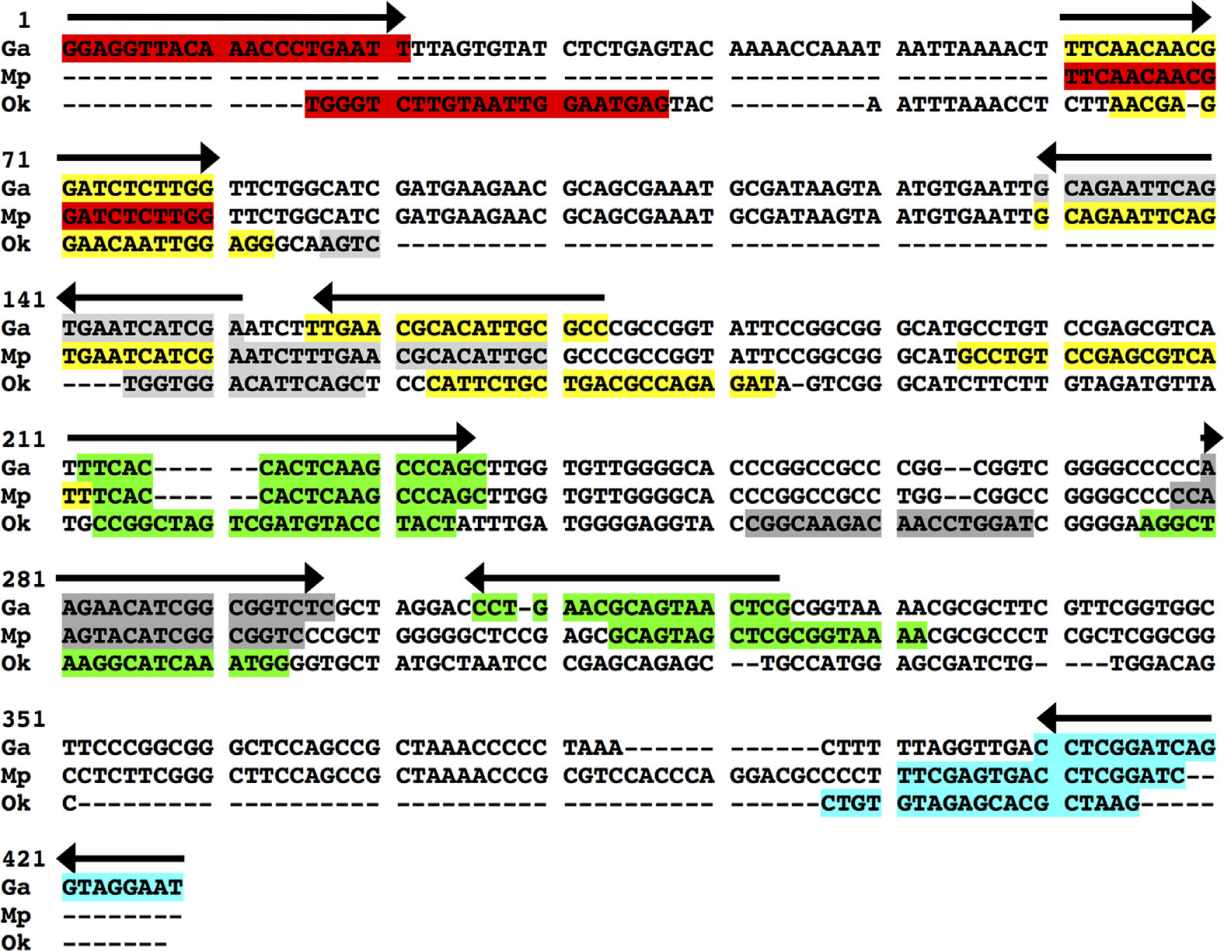
Sequence alignment of the three fungi for the F3-B3 PCR amplicon for the loop-mediated isothermal amplification (LAMP) primer sets of *Magnaporthiopsis poae* (Mp), *Ophiosphaerella korrae* (Ok), and *Gaeumannomyces avenae* (Ga). Sequence coloring for primer nucleotide locations red equals F3, sky blue equals B3, yellow equals FIP (F2 and F1c), green equals BIP (B2 and B1c), light grey equals LoopF, dark grey equals LoopB. Arrows show directions only for Ga primer set and the directions are the same for Mp and Ok primer sets as well.

**Fig. 2:**
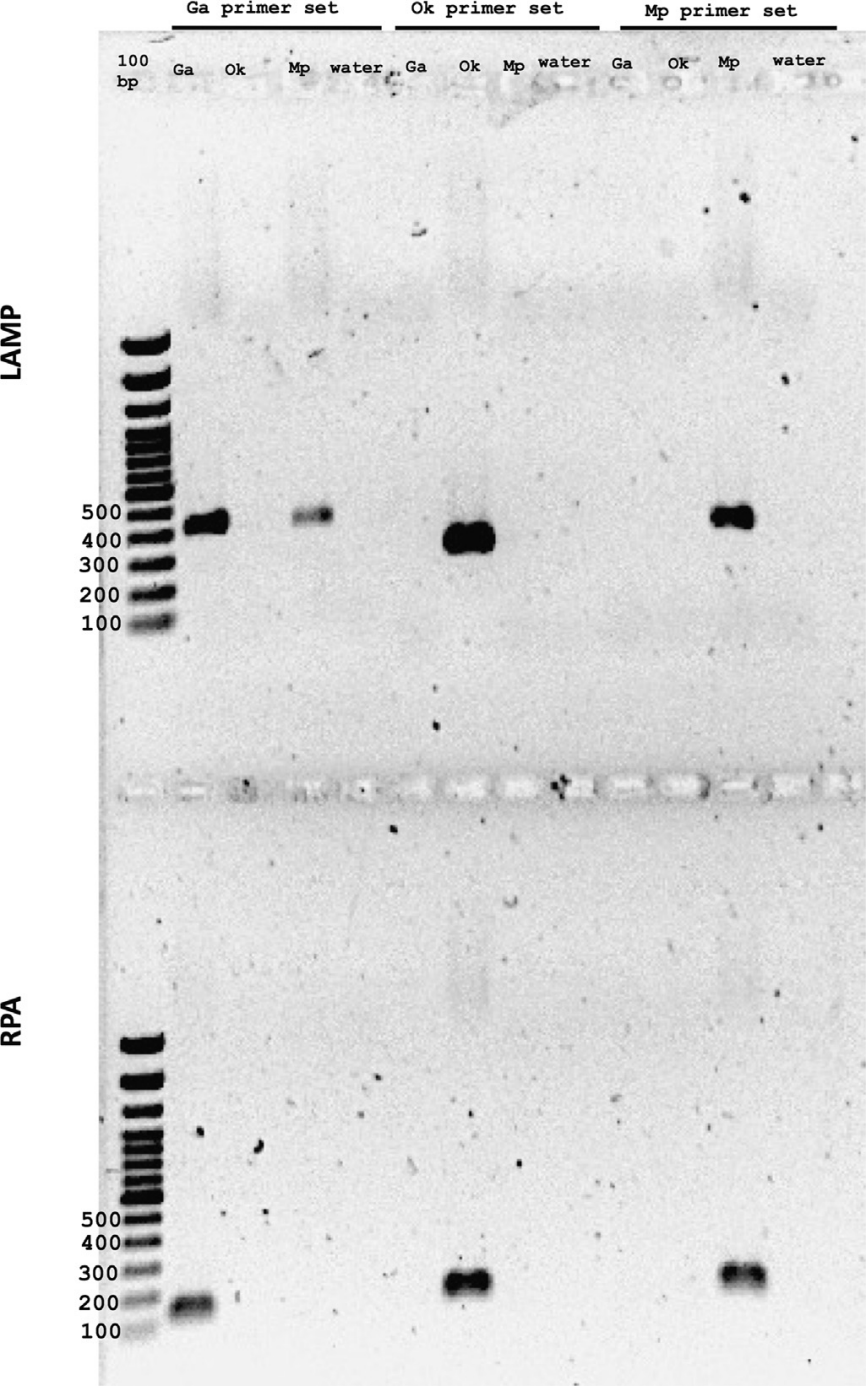
PCR amplicons for the loop-mediated isothermal amplification (LAMP) and recombinase polymerase amplification (RPA) primer sets of *Magnaporthiopsis poae* (Mp), *Ophiosphaerella korrae* (Ok), and *Gaeumannomyces avenae* (Ga). For LAMP primer sets, Mp F3-B3 amplicon size equals 350 bp, Ok F3-B3 amplicon size equals 284 bp, and Ga F3-B3 amplicon size equals 407 bp. For the RPA primer sets, Mp amplicon size equals 129 bp, Ok amplicon size equals 141 bp, and Ga amplicon size equals 124 bp.

**Fig. 3:**
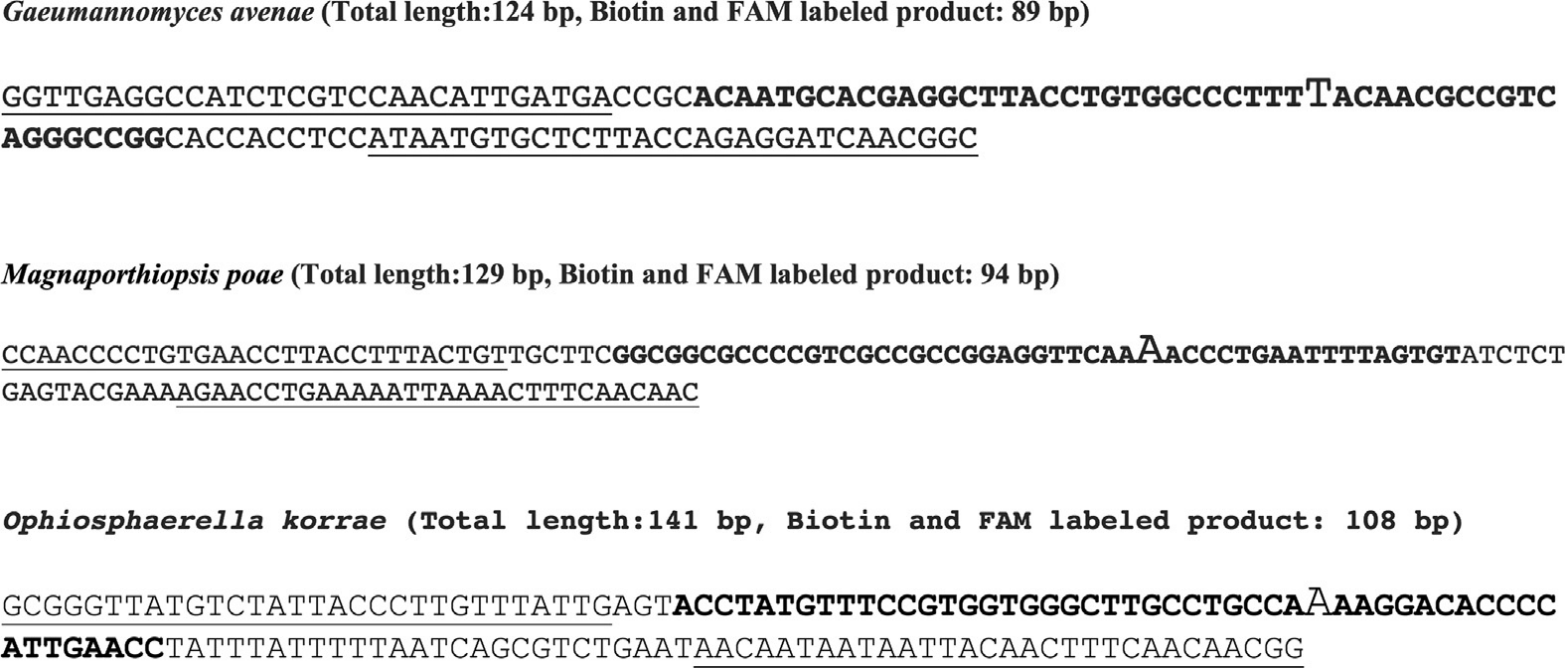
Recombinase polymerase amplification (RPA) primer/probe nucleotides for the three fungi. The underlined sequences at 5′ and 3′ end are the forward and reverse primers, respectively. The probe is shown in bold with the nucleotide where the probe is cleaved to form the shorter FAM and Biotin labeled product shown in the larger size. The total length and the RPA product generated by probe and reverse primer detection are shown.

The sequences between the F3 and B3 primers is the largest product formed in a LAMP reaction. In *Gaeumannomyces avenae* and *Magnaporthiopsis poae* these sequences share a very high homology (86% identity), but both *G. avenae* and *M. poae* had a lesser homology with *Ophiosphaerella korrae* in this region (45% and 43% identity, respectively) (Fig. 1). Sequenced PCR amplicons of positive control fungal genomic DNA with either the F3-B3 LAMP primers or forward and reverse primers of RPA resulted in 99% identity for each respective fungus when queried in BLASTn. The specific 50 base acceler8™ probe sequence for RPA identified only the respective fungi when queried on BLASTn. For both LAMP and RPA, the primers designed for one fungus did not amplify any other genome except in one case where LAMP primers for *G. avenae* amplified *M. poae* using *M. poae* genomic DNA (Fig. 2). The carboxyfluorescein (FAM) and biotin labeled RPA products for *G. avenae*, *M. poae* and *O. korrae* were 89 bp, 94 bp and 108 bp, respectively (Fig. 3). Both assays were also tested for false positives that can occur with root-infecting pathogens other than *G. avenae*, *M. poae* and *O. korrae* [1].

### 1.2 Field sample evaluation by RPA

**Fig. 4:**
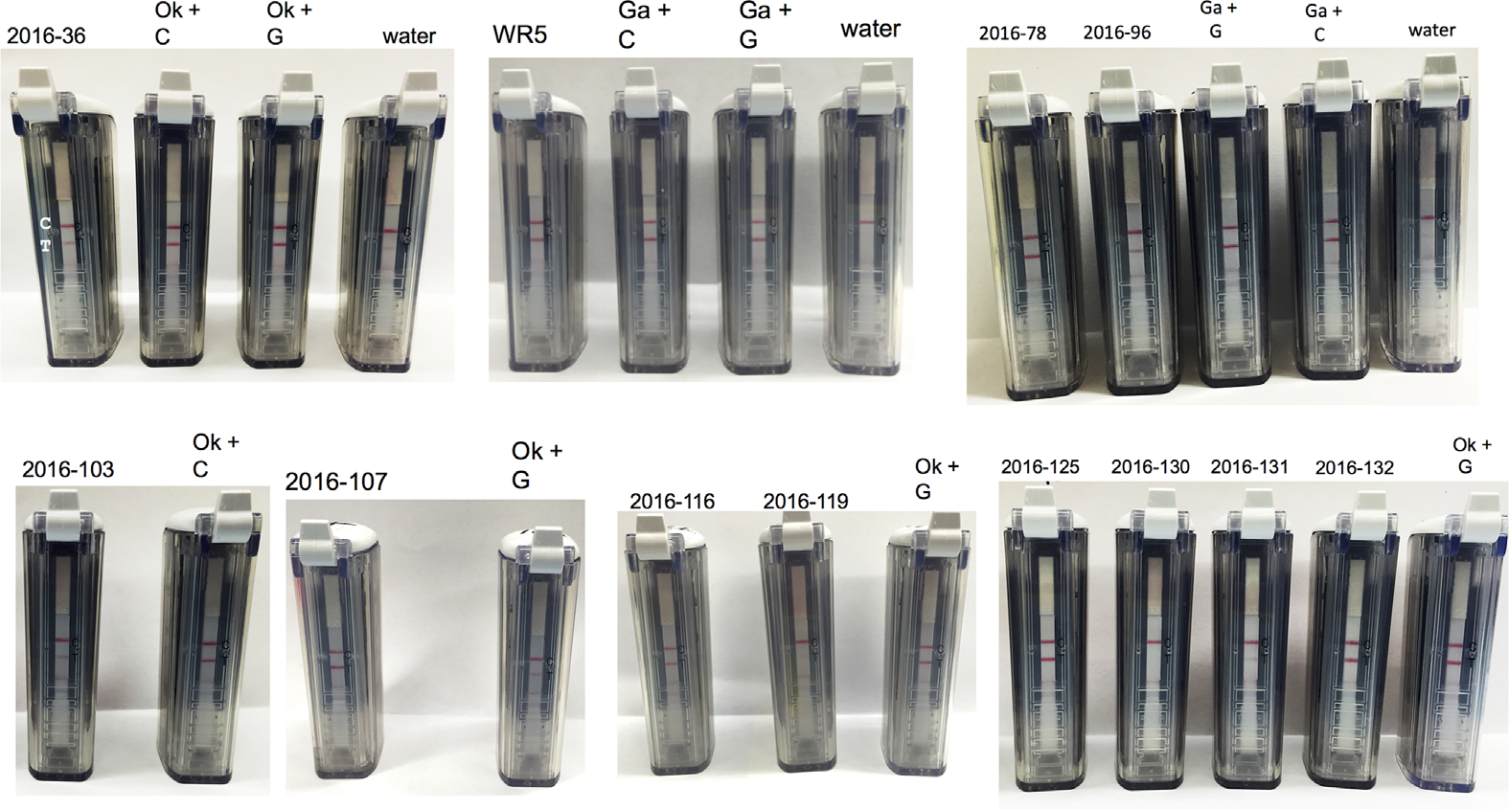
Recombinase polymerase amplification (RPA) on 12 root samples in 2016 from different cool-season turfgrass species diagnosed for *Magnaporthiopsis poae* (Mp), *Ophiosphaerella korrae* (Ok), and *Gaeumannomyces avenae* (Ga). Assay was performed with positive controls of (+) fungal culture extract (C) or genomic DNA (G) for Mp, Ok, and Ga.

RPA assay was used to test 12 samples in 2016 (Fig. 4). Three creeping bentgrass (*Agrostis stolonifera*) samples (2016-78, 2016-96, WR5) were diagnosed as take-all patch using symptom morphology and microscopic inspection and the RPA- *G. avenae* assay detected *G. avenae* on the roots of both samples. Six Kentucky bluegrass (*Poa pratensis*), samples (2016-36, 2016-103, 2016-116, 2016-119, 2016-125, 2016-132) and one annual bluegrass (*P. annua*) sample (2016-107) were diagnosed as necrotic ring spot using visual characteristics and microscopy, and the RPA-*O. korrae* assay detected *O. korrae* on all seven samples. Kentucky bluegrass samples 2016-130 and 2016-131 were diagnosed as *O. korrae* using microscopy but *O. korrae* was not detected with the RPA-*O. korrae* assay (Table 1 from [1]). We did not receive any samples diagnosed as summer patch in 2016 and therefore, the RPA-*M. poae* assay was not used.

**Table 1:** LAMP primers and RPA primers and probes for the three fungi.

		**Name**	**Primer or probe**	**Position**	**Orient**	**Length**	**Tm**	**GC%**
*Gaeumannomyces avenae*	LAMP	GGA_AY428778_F3	GGAGGTTACAAACCCTGAATT	128–148	→	21	60.2	42.9
		GGA_AY428778_B3	ATTCCTACCTGATCCGAGG	516–534	←	19	60.0	52.6
		GGA_AY428778_FIP	GGCGCAATGTGCGTTCAATTCAACAACGGATCTCTTGG	283–300+188–207	←+→	38	40.9	53.0
		GGA_AY428778_BIP	TTCACCACTCAAGCCCAGCCGAGTTACTGCGTTCAGG	339–357+425–442	→+←	37	66.8	50.0
		GGA_AY428778_LoopF	TCGATGATTCACTGAATTCTGC	256–278	←	22	61.1	40.9
		GGA_AY428778_LoopB	AAGAACATCGGCGGTCTC	399–416	→	18	61.8	55.6
	RPA	U17568_TAP_FOR	GGTTGAGGCCATCTCGTCCAACATTGATGA	187–217	→	30	63.4	50.0
		U17568_TAP_REV^a^	/5Biosg/GCCGTTGATCCTCTGGTAAGAGCACATTAT	281–310	←	30	61.2	46.7
		TAP_probe_Acceler8	/56-FAM/ACAATGCACGAGGCTTACCTGTGGCCCT TT/idSp/ACAACGCCGTCAGGGCCGG/3SpC3/	281–310	→	30	61.2	46.7
*Magnaporthiopsis poae*	LAMP	F3_MAG3-2	TTCAACAACGGATCTCTTGG	180–199	→	20	60.2	45
		B3_MAG3-2	GATCCGAGGTCACTCGAA	512–529	←	18	60.5	55.6
		FIP_MAG3-2	AATGACGCTCGGACAGGCGCAGAATTCAGTGAATCATCG	314–331+249–269	←+→	39	51.2	67.7
		BIP_MAG3-2	TCACCACTCAAGCCCAGCTTTACCGCGAGCTACTGC	332–349+425–442	→+←	36	58.3	69.6
		LoopF_MAG3-2	GCAATGTGCGTTCAAAGATT	270–289	←	20	60.3	40.0
		LoopB_MAG3-2	CCAAGTACATCGGCGGTC	370–389	→	18	62.6	61.1
	RPA	JX134596_SP_FOR	CCAACCCCTGTGAACCTTACCTTTACTGTT	72–101	→	30	46.7	61.6
		JX134596_SP_REV^a^	/5Biosg/GTTGTTGAAAGTTTTAATTTTTCAGGTTCT	171–200	←	30	64.0	35.7
		SP_probe_Acceler8^b^	/56-FAM/GGCGGCGCCCCGTCGCCGCCGGAGGTT CAA/idSp/ACCCTGAATTTTAGTGT/3SpC3/	107–154	→	48	54.0	26.7
*Ophiosphaerella korrae*	LAMP	F3_NRS_KP690981	TGGGTCTTGTAATTGGAATGAG	401–422	→	22	60.4	40.9
		B3_NRS_KP690981	CTTAGCGTGCTCTACACAG	666–684	←	19	60.0	52.6
		FIP_NRS_KP690981	ATCTCTGGCGTCAGCAGAATGAACGAGGAACAATTGGAGG	484–504+440–458	←+→	40	67.3	50.0
		BIP _NRS_KP690981	CCGGCTAGTCGATGTACCTACTCCATTTGATGCCTTAGCCT	533–554+595–614	→+←	41	67.2	51.2
		LoopF_MAG3-2_NRS_KP690981	GCTGAATGTCCACCAGACT	461–480	←	19	61.8	52.6
		LoopB_MAG3-2_NRS_KP690981	CGGCAAGACAACCTGGAT	572–589	→	18	62.1	55.6
	RPA	U04862_NRS_FOR	GCGGGTTATGTCTATTACCCTTGTTTATTG	95-12	→	30	57.5	40
		U04862_NRS_REV^a^	/5Biosg/CCGTTGTTGAAAGTTGTAATTATTATTGTT	205–235	←	30	53.6	26.7
		NRS_probe_Acceler8^b^	/56-FAM/ACCTATGTTTCCGTGGTGGGCTTGCCTG	128–178	→	50	57.5	40
			CCA/idSp/AAGGACACCCCATTGAACC/3SpC3/					

Loop mediated isothermal amplification (LAMP) primers were designed on Optigene® ‘s LAMP designer software from GenBank No. AY428778 for *Gaeumannomyces graminis v*ar. *avenae*, GenBank No. JX134597 for *Magnaporthe poae* and GenBank No. KP690981 for *Ophiosphaerella korrae*. Recombinase polymerase amplification (RPA) primers and probes were designed manually based on Agdia® AmplifyRP® acceler8™’s instructions from GenBank No. U17568 for *G. graminis v*ar. *avenae*, GenBank No. JX134596 for *M. poae* and GenBank No. U04862 for *O. korrae*.

a All the RPA reverse primers have Biotin label at the 5′ end.

b The three RPA fungal sequence probes have 6-Carboxyfluorescein (56-FAM) at the 5′ end, an abasic nucleotide analog dSpacer,1,2′-Dideoxyribose (idSp) replaces a nucleotide internally and polymerase extension blocking group C3 Spacer phosphoramidite (3SpC3) at the 3′ end.

## 2 Experimental design, materials and methods

### 2.1 LAMP primer design

The LAMP primers were designed using LAMP Designer Windows 7 program version (PREMIER Biosoft Palo Alto, CA) and the six primer nucleotides (F3, B3, FIP, BIP, LoopF and Loop B) were synthesized by Integrated DNA Technologies in Coralville, IA. The 18S ribosomal genes were selected for primer design for the three fungi (Table 1). The *G. avenae* primer was developed using GenBank sequence number AY428778 isolated from an unknown plant source in a phylogenetic study by [5]. The *M. poae* primer was developed using GenBank number JX134597 isolated from annual bluegrass roots [2] and the *O. korrae* primer was developed using GenBank sequence number KP690981 from an isolate collected from Kentucky bluegrass roots [4]. Amplicons were generated from F3 and B3 on positive genomic DNA from fungal culture controls by PCR and sequenced. Further descriptions of the LAMP assay can be found in [1].

### 2.2 RPA Accler8 primer-probe design

Primers and probes were designed using Agdia® AmplifyRP® (Elkhart, IN) acceler8^™^ instructions and synthesized by Integrated DNA Technologies. The primer sets for *G. avenae* were designed using the gene coding for *avenacinase* (GenBank No. U17568) from an isolate collected from oats (*Avena sativa*) [3]. The primer sets for *M. poae* amplified the 18S ribosomal RNA gene (GenBank No. JX134596) from an isolate obtained from annual bluegrass [2]. The primer sets for *O. korrae* also amplified the 18S ribosomal RNA gene (GenBank No. U04862) from an isolate obtained from Kentucky bluegrass roots [6].

Before synthesizing probes and modifying the reverse primer with antigen labels, PCR and sequencing were conducted as described for LAMP using forward and reverse primers on genomic DNA of positive fungal cultures to confirm amplicon specificity. The labels on probes and reverse primers are shown in Table 1. The reverse primer has a biotin antigen label attached to the 3′ end. The accler8^™^ probes for each fungus were internal to the two primers and were in reverse orientation to the reverse primer. Each probe had a 5′ FAM, an abasic nucleotide analog dSpacer,1,2′-Dideoxyribose (idSp) replacing a nucleotide internally (Table 1) to create a mismatch to be recognized by endonuclease IV (nfo) when DNA polymerase synthesizes a new sequence, and polymerase extension blocking group C3 Spacer phosphoramidite (3SpC3) at the 3′ end. Further descriptions of the RPA assay can be found in [1].

### 2.3 RPA assay validation on field samples

The samples from 2016 were evaluated by RPA as described in section 2.6 of [1]. Briefly, root extracts were prepared by grinding soil-free 30–40 fungal-infested roots (examined by presence of melanized hyphae under microscope) in 0.2 N sodium hydroxide. Each sample reaction was conducted in a sterile nuclease-free 1.7-ml microcentrifuge tubes containing 5.9 µl of rehydration buffer, 0.50 µl of 280 mM magnesium acetate (both provided by acceler8^TM^), 0.42 µl of 10 µM forward and reverse primers, 0.12 µl of 10 µM accler8 probe, 1 µl of root extract and 1.64 µl of PCR-grade nuclease-free sterile water. The tubes were vortexed and spun briefly and the solution was transferred into 0.2-ml microcentrifuge tube in the acceler8^™^ kit and thoroughly suspended in a white reaction pellet present in the tube. The capped 0.2 ml-tubes were vortexed and spun briefly and incubated for 20 min at 39 °C in water bath (Isotemp 205, Fisher Scientific, Waltham, MA). The incubated tubes with labeled amplicons were transferred to a plastic apparatus containing proprietary solution for biotin and FAM antigen-antibody reactions. The apparatus was transferred to a detection chamber housing an immunodetection paperstrip that has anti-FAM and anti-biotin label lines towards the center. When the reaction tubes are snapped shut in the detection chamber, the RPA reaction tubes and the proprietary solution rises on to the paperstrip by lateral flow. The reaction has worked if a black control (C) line appears towards the center of the paper strip and a test (T) black line appears below C line if the specific fungal DNA is present.

The RPA results, sample location, grass type and microscopy diagnosis of the 2016 samples are listed in Table 1 of [1] along with LAMP and RPA on samples from 2017.

## Supplementary material

Transparency document
