# Supplementary material for: Data for designing two isothermal amplification assays for the detection of root-infecting fungi on cool-season turfgrasses
Source: Data Brief. 2018 Aug 17;20:471–9. doi: 10.1016/j.dib.2018.08.021 (PMC6122334; doi:10.1016/j.dib.2018.08.021)
Supplement: Supplementary file 1 — Transparency document [file mmc1.pdf]

THE UNIVERSITY of WISCONSIN MADISON

July 9th, 2018

Journal of Microbiological Methods – Data in Brief

The authors declare they have no conflicts of interest in the publication of this manuscript or in the research described within.

Sincerely,

Brijesh Karakka, Ph.D. and Paul Koch, Ph.D.
Department of Plant Pathology
University of Wisconsin - Madison

The Koch Lab   Department of Plant Pathology
College of Agricultural and Life Sciences   Russell Laboratories
University of Wisconsin-Madison
1630 Linden Drive Room 283 Madison, Wisconsin 53706   608/262-6531   Fax: 608/263-2626
